# The Role of Psychiatric, Analgesic, and Antiepileptic Medications in Chronic Pruritus

**DOI:** 10.7759/cureus.17260

**Published:** 2021-08-17

**Authors:** Parth Patel, Keshav Patel, Karan Pandher, Ruqiya Shama Tareen

**Affiliations:** 1 Dermatology, Lake Erie College of Osteopathic Medicine, San Diego, USA; 2 Internal Medicine, University of Illinois Chicago, Kalamazoo, USA; 3 Dermatology, Rosalind Franklin University Chicago Medical School, North Chicago, USA; 4 Psychiatry, Western Michigan University Homer Stryker MD School of Medicine, Kalamazoo, USA

**Keywords:** somatosensory processing, chronic pruritus, psychogenic pruritus, medications, refractory

## Abstract

Chronic pruritus is a complex yet prevalent concern without a gold standard treatment. The mainstay therapy for chronic pruritus includes topical ointments such as corticosteroids, capsaicin, local anesthetics, antihistamines, and immunomodulators. There are many different subtypes of chronic pruritus, and each unique subtype may benefit from specialized treatments. This review article sheds light on the role of psychiatric, analgesic, and antiepileptic medications in chronic pruritus. We believe that further large-scale studies are needed to determine the true effectiveness of these medications in treating chronic pruritus.

## Introduction and background

Pruritus is a prevalent complaint in both primary care and dermatology clinics. Many patients experience intermittent pruritus; however, chronic pruritus is reserved for individuals with a sensation of itching for more than six weeks [1]. An estimated 8% of the adult population suffers from chronic itch [2]. A deeper examination into the underlying causes of pruritus shows that systemic diseases, inflammatory skin diseases, external factors, genetics, and endogenous factors can be the underlying causes of itch [3]. Because itch is a complex complaint, the diagnostic workup should be exhaustive and include a complete blood count with differential, sedimentation rate, C-reactive protein, creatinine and glucose levels, liver function tests, thyroid function tests, and can even include further tests beyond these preliminary ones [3]. If these tests do not reveal an underlying cause for a patient’s chronic itch, diagnoses of exclusion should be considered.

It is important to consider the different somatosensory processing involved in the perception of itch and differentiate it from other sensations to better understand the different pathways to target itch. A person perceives and distinguishes the type of sensation caused by a stimulus as producing discomfort, pruritus, or pain and assigns a level of intensity to that stimuli. This makes that particular sensation qualitatively different for that person compared to any other person. Once this stimulus is perceived by the brain as belonging to a specific modality, it is identified and cataloged. When challenged again with a similar stimulus, the specific response is generated based on previous experiences. “Metaesthetic sensation,” a term used by Stante et al. [4], is described as a continuum of sensation starting on one end as a sensation of touch to the other end of the spectrum as a sensation of pain. This concept is important as a better understanding of the itch and other sensory pathways may help develop better treatments. In addition, patients suffering from one type of chronic sensory stimulation, such as chronic pruritus, may also be sensitive to another type of sensory stimulation, and it is likely that chronic pruritus patients may have a lower threshold to pain and vice versa [5,6]. This seems apparent in patients with shingles, a herpes zoster reactivation infection, who may develop both postherpetic neuralgia (PHN) and chronic postherpetic itch (PHI) [7]. Because there are various variables to influence the classification and perception of chronic stimuli to be a chronic itch, providers may treat each patient individually utilizing a case-by-case approach.

The mainstay therapy of chronic pruritus includes topical ointments such as corticosteroids, capsaicin, local anesthetics, antihistamines, immunomodulators, cannabinoids, and salicylic acid [8]. However, many other medications such as psychiatric, analgesic, and antiepileptic medications have also been successful in treating chronic pruritus [8]. This review article sheds light on the role of psychiatric, analgesic, and antiepileptic medications in chronic pruritus.

## Review

Psychiatric medications

Pruritus is often thought of as a dermatologic problem, but in many patients, it may result from a primary psychogenic illness. In a study in which patients met the Diagnostic and Statistical Manual of Mental Disorders-IV criteria for schizophrenic, affective, and other psychiatric disorders, 32% of the participants had a chronic itch [2]. Further studies are required to better elucidate the relationship between pruritus and psychiatric disorders. Multiple subtypes of psychogenic chronic pruritus exist [9]. The three main subtypes include a primary dermatologic disorder with pruritus that leads to a psychiatric condition, stress-induced exacerbation of the dermatologic disease, and a primary psychiatric condition that leads to pruritus [8,9]. For example, patients diagnosed with depression often develop pruritus that is exacerbated by trauma, and animal studies in depressed mice have demonstrated a causal relationship between depression and chronic itch [8]. In the next few paragraphs, we will discuss the utility of selective serotonin reuptake inhibitors (SSRIs), serotonin and norepinephrine reuptake inhibitors (SNRIs), atypical antidepressants, tricyclic antidepressants (TCA), and antipsychotics in chronic pruritus (see Table 1).

**Table 1:** Psychiatric medication use in chronic pruritus. DOP: delusions of parasitosis

Medication	Authors	Type of study	Number of patients in the study	Pruritus association
Mirtazapine	Hundley et al. (2004) [10]	Uncontrolled novel	3	Nocturnal pruritus
Mirtazapine	Khanna et al. (2019) [11]	Systematic review	N/A	Atopic dermatitis, lichen simplex chronicus, advanced renal cell carcinoma, Hodgkin’s lymphoma, large B-cell lymphoma, adenocarcinoma
Paroxetine/Fluvoxamine	Ständer et al. (2009) [12]	Open-labeled study	72	Atopic dermatitis, systemic lymphoma, solid carcinoma
Sertraline	Mayo et al. (2007) [13]	Double-blinded, placebo-controlled	N/A	Chronic liver disease
Doxepin	Eschler and Klein (2010) [14]	Systematic review	N/A, 19 trials	General, not specified
Amitriptyline	Magazin et al. (2019) [15]	Case report	1	Brachioradial pruritus
Apriprazole	Ladizinski et al. (2010) [16]	Case study	8	DOP
Pimozide	Lorenzo et al. (2004) [17]	N/A	N/A	DOP
Olanzapine	Meehan et al. (2006) [18]	Case study	3	DOP

Selective Serotonin Reuptake Inhibitors

SSRIs are commonly used as a first-line medication for depression. SSRIs can also be used for generalized anxiety disorder, obsessive-compulsive disorder, social anxiety disorder, and bulimia nervosa. Some common SSRIs include citalopram, escitalopram, fluoxetine, paroxetine, fluvoxamine, and sertraline. As their name implies, SSRIs inhibit the reuptake of 5-hydroxytryptamine (5-HT).

SSRIs can also be an effective therapy for chronic pruritus. In an open-label study involving 72 patients, paroxetine and fluvoxamine reduced pruritus associated with atopic dermatitis, systemic lymphoma, or solid carcinoma in 68% of patients [12]. Sertraline is particularly effective in patients with pruritus secondary to chronic liver disease [1]. A double-blinded, placebo-controlled trial found that 75-100 mg/day of sertraline improved itch scores significantly in such patients [13]. One systematic review found eight randomized controlled trials (RCTs) which demonstrated that the use of SSRIs significantly reduced chronic itch in patients [19]. One of the RCTs included 25 patients with either solid or hematologic malignancies that caused an itch. Out of the 25 patients who were given 20 mg of the SSRI paroxetine once every morning for a week, 37.5% reported >50% reduced itch from the baseline [19].

Further, it has been hypothesized that SSRIs reduce itch through the attenuation of serotonergic signals, which provide inhibitory inputs to itch pathways in the central nervous system [13]. Specifically, the eventual downregulation of central 5-HT3 receptors from repetitive stimulation leads to the antipruritic effect [12]. Peripherally, it has been proposed that, through an unspecified mechanism, SSRIs diminish nociceptive stimulus transmission through unmyelinated C fibers that produce the sensation of itch [19].

In addition to the well-established analgesic effect of certain antidepressants, SSRIs and other antidepressants are effective in treating anxiety and depression in many patients with chronic pain conditions. Because the high comorbidity of depression and anxiety in patients suffering from chronic pain and related conditions such as pruritus has been well established, it will be prudent to screen for depression and anxiety in these patients and treat them with antidepressants if indicated.

Serotonin and Norepinephrine Reuptake Inhibitors

SNRIs can be used to treat depression, anxiety, fibromyalgia, diabetic peripheral neuropathy, and chronic musculoskeletal pain. Some common SNRIs include desvenlafaxine, duloxetine, levomilnacipran, milnacipran, and venlafaxine. As their name implies, SNRIs inhibit the reuptake of serotonin (5-HT) and norepinephrine. SNRIs decrease the perception of pruritus in the cerebral cortex by interfering with norepinephrine and serotonin transmission. In a case report of a 75-year-old woman with treatment-resistant prurigo for three months with a Visual Analog Scale (VAS) pain score of 96 out of 100 for pruritus, 20 mg of duloxetine hydrochloride per day was given for one week. The patient reported a score of zero on the VAS scale with complete resolution of the itch [20]. In a second case report with a 61-year-old woman with treatment-resistant prurigo for three months with a VAS score of 100, administering 20 mg of duloxetine hydrochloride for one week resulted in complete resolution of her itch and a VAS score of zero, indicating full resolution of the itch [20]. For reference, the term “treatment-resistant” in both of these cases was defined as patients not responding to the traditional topical corticosteroids or antihistamines for the treatment of their prurigo [20].

Duloxetine is a newer and unique agent as it produces an analgesic effect independent of its antidepressant effect. It inhibits the reuptake of serotonin and norepinephrine more specifically in the descending inhibitory spinal pathways and reduces the nociceptive afferent transmission in the ascending spinal pain pathways [21]. Thus, the analgesic effect of duloxetine is independent of the presence or absence of depression. The analgesic effect of duloxetine is achieved at lower doses and within few days, which is in contrast to four to six weeks of treatment on higher doses to achieve a reasonable antidepressant effect [20,21].

Atypical Antidepressants

Atypical antidepressants are used for atypical or refractory depression. Atypical antidepressants include bupropion and mirtazapine. Mirtazapine increases norepinephrine through the antagonism of alpha-2 autoreceptors and alpha-2 heteroreceptors and increases serotonin transmission by the antagonism of 5-HT2 and 5-HT3 reuptake receptors. Mirtazapine is also an agonist at 5-HT1A postsynaptic receptors and has been known to antagonize histaminergic H1 receptors. It has been hypothesized that mirtazapine reduces itch through central alpha-2 receptor antagonism along with histamine and serotonergic antagonism [10].

Mirtazapine has also shown efficacy in modulating the opioid-mediated analgesic effect. Prolonged antidepressant administration has been shown to induce endogenous opioid release and modify opioid receptors density, potentiating the analgesic effect. Mirtazapine and other antidepressants can also have an antineuropathic pain effect through an antagonistic effect on N-methyl-D-aspartate [22].

According to an uncontrolled novel study including three patients with severe nocturnal pruritus, mirtazapine was found to be an effective treatment option for nocturnal pruritus [10]. In a systematic review, 15 mg of mirtazapine was effective in treating chronic pruritus associated with atopic dermatitis, lichen simplex chronicus, adenocarcinoma, Hodgkin’s lymphoma, large B-cell lymphoma, and advanced renal cell carcinoma that were refractory to first-line treatment [11].

Tricyclic Antidepressants

TCAs have been used effectively to treat depression for several decades and were the mainstay of treatment for depression until newer antidepressants in the form of SSRIs became the first line of treatment. TCAs are still used for depression that is refractory to first-line medications or in situations where comorbid conditions such as insomnia, pruritus, or pain require a different approach. They are also effective in treating the pain associated with chronic pruritus. TCAs exert this effect by decreasing the reuptake of both serotonin (5-HT) and norepinephrine. Both of these neurotransmitters are instrumental in providing itch relief by affecting pain and other somatosensory pathways at the central and peripheral levels. Norepinephrine provides itch relief via actions on α-1 and α-2 adrenoceptors, and serotonin provides itch relief via actions on 5HT-1 through 4 receptors. TCAs also exert an inhibitory effect on cholinergic, muscarinic, and histamine receptors, which have antinociceptive properties. The antihistamine and anticholinergic action of antidepressants may also facilitate pain signaling [15].

Nowadays, TCAs are more commonly used for insomnia, peripheral neuropathy, chronic pain, and nocturnal enuresis rather than for depression. Amitriptyline is the most commonly used TCA for analgesic purposes. Amitriptyline also has a unique analgesic action through the activation of the adenosine A1 receptors. Some other commonly used TCAs are desipramine, doxepin, imipramine, nortriptyline, and clomipramine.

TCAs such as doxepin, amitriptyline, and nortriptyline have been effective in alleviating itch [23]. According to a systematic review of 19 clinical trials, topical doxepin given in low doses, defined as 10-100 mg, was effective in treating chronic pruritus [3,14]. In a case report on a patient with refractory brachioradial pruritus, a combination of topical amitriptyline and ketamine proved effective [15].

Pruritus relief by TCAs is hypothesized to occur through the inhibition of 5-HT and norepinephrine reuptake on cutaneous nerves. This reduces the sensation of itch, more specifically the burning and tingling symptoms [15]. Given the established use of TCAs for peripheral neuropathy, this seems to be a logical explanation for its mechanism of action. In addition, many TCAs have antihistamine properties.

Antipsychotics

Antipsychotics are used to treat certain pathologies such as schizophrenia, bipolar disorder, Tourette’s syndrome, and Huntington’s disease. There are two categories of antipsychotics, namely, typical and atypical. Typical antipsychotics mainly block dopamine D2 receptors. Atypical antipsychotics have a wide range of mechanisms. However, most of these medications primarily block 5-HT2 and dopamine D2 receptors.

Antipsychotics are the drug of choice in treating chronic pruritus secondary to delusions of parasitosis (DOP) [9]. Pimozide, a typical antipsychotic, inhibits dopamine D2, alpha-adrenergic, and 5-HT2 receptors [24]. It is the treatment of choice for DOP [17]. Aripiprazole, an atypical antipsychotic, is a partial agonist at dopamine D2 and serotonin 5-HT1A receptors [25]. This medication is also an antagonist at the serotonin 5-HT2A receptor [25]. Aripiprazole is an effective treatment for DOP according to a study in which eight DOP patients were followed for six months, all of whom experienced complete remission of their symptoms [16]. Olanzapine, an atypical antipsychotic, inhibits dopamine D2 and 5-HT2A receptors. In a recent case study of three patients with DOP, 5 mg of olanzapine daily was successful in treating the disease [18].

Delusions originate from the positive symptoms of dopamine excess through the mesolimbic pathway in the brain, which contains an abundance of D2 receptors, playing the largest role in producing delusions [17,18]. Therefore, the mechanism of antagonists of D2 receptors providing pruritic relief is feasible to minimize the production of these delusions through antagonism of these receptors [17,18].

Analgesics/Opioids

Opioids can also be used for chronic pruritus (Table 2). μ-opioid receptor antagonists and k-opioid receptor agonists reduce itch [26]. Naltrexone, a μ-opioid antagonist, reduced pruritus in patients with cholestasis, atopic dermatitis, burns, and end-stage renal disease [27-30]. In a single-blinded, self-controlled trial involving 34 patients with cholestatic pruritus, naltrexone showed a significant decrease in daytime and nighttime pruritus in 38% of patients [27]. In a double-blinded, placebo-controlled trial, patients with atopic dermatitis-associated itch were administered 50 mg daily doses of naltrexone for two weeks which resulted in markedly reduced itch [28]. In another study, 19 burn victims who were experiencing pruritus were given naltrexone for two weeks resulting in a significant decrease in itching sensation [29]. In a randomized, double-blind, placebo-controlled, crossover trial, 15 patients with chronic pruritus due to end-stage renal disease were given 50 mg of naltrexone per day, which resulted in significantly reduced itch [30]. Another opioid showing promise in the treatment of chronic pruritus is nalmefene, a μ-opioid antagonist. Nalmefene reduced itch in chronic pruritus caused by cholestasis by 75% in a double-blinded study involving 14 patients [31]. K-opioid agonists such as butorphanol and nalfurafine are also effective for chronic pruritus [9]. Intranasal butorphanol was administered in a case series of five patients and showed significant improvement in patients with intractable pruritus due to dermatologic and systemic diseases [32]. In a phase III, double-blinded study with 337 patients, 5 mg and 2.5 mg of nalfurafine resulted in a significant reduction in itch compared to the placebo [33]. The placebo group included 111 patients, the 2.5 mg group included 112 patients (P = 0.0001), and the 5 mg group included 114 patients (P = 0.0002) [33].

**Table 2:** Opioid use in chronic pruritus.

Medication	Authors	Type of study	Number of patients in the study	Pruritus association
Naltrexone	Mansour-Ghanaei et al. (2006) [27]	Single-blinded, self-controlled	34	Cholestatic pruritus
Naltrexone	Malekzad et al. (2009) [28]	Double-blinded, placebo-controlled	N/A	Atopic dermatitis
Naltrexone	Jung et al. (2009) [29]	Case study	19	Burn victims
Naltrexone	Peer et al. (1996) [30]	Randomized, double-blinded, placebo-controlled	15	End-stage renal disease
Nalmefene	Bergasa et al. (1999) [31]	Double-blinded	14	Cholestasis
Butorphanol	Dawn and Yosipovitch (2006) [32]	Case series	5	Dermatologic diseases and systemic diseases
Nalfurafine	Kumagai et al. (2010) [33]	Phase III, double-blinded	337	N/A

Anticonvulsants

Neuroepileptics may have the most promise among the psychogenic medications for the treatment of certain subsets of chronic pruritus (Table 3). Gabapentin, a structural analog to the neurotransmitter gamma-aminobutyric acid (GABA), is an anticonvulsant drug that exerts its actions by inhibiting voltage-gated calcium channels in the presynaptic cleft to inhibit the release of neuronal neurotransmitters. Regarding pruritus, it has been hypothesized that this can inhibit the release of neurotransmitters in the neural pathway of itching sensation [34]. Gabapentin was an effective therapy for chronic pruritus secondary to chronic renal failure in 24/25 patients in a double-blinded, placebo-controlled trial (P = 0.0001) [34]. In a case study involving two patients with chronic itch associated with unknown origin, treatment with gabapentin was successful [35]. The dosing was started at 300 mg/day and gradually increased to 1,800 mg/day with complete resolution of itch within one month of treatment [35]. In a case study, pregabalin, another GABA analog, was beneficial in treating all three patients with chronic pruritus, resulting in a reduction of itch by over 70% using VAS for pain index [36]. Pregabalin has the same mechanism of action as gabapentin in decreasing presynaptic neural neurotransmitter release as well as neural excitation downstream. Both pregabalin and gabapentin can be hypothesized as decreasing chronic pruritus by reducing the central sensitization of neurons involved in the generation of chronic pruritus [36]. Both pregabalin and gabapentin provide an added advantage of addressing anxiety associated with most chronic pain conditions. However, side effects of excessive sedation and weight gain may limit their use in some patients. Valproate may also be beneficial for chronic pruritus. In one study of 14 patients, combination treatment of hydralazine and valproate was effective in reducing pruritus in cutaneous T-cell lymphoma patients [37].

**Table 3:** Anticonvulsant use in chronic pruritus.

Medication	Authors	Type of study	Number of patients in the study	Pruritus association
Gabapentin	Gunal et al. (2004) [34]	Double-blinded, placebo-controlled	25	Chronic renal failure
Gabapentin	Yesudian and Wilson [35]	Case study	2	Unknown origin
Pregabalin	Ehrchen et al. (2008) [36]	Case study	3	N/A
Valproate (combined with hydralazine)	Espinoza-Zamora et al. (2017) [37]	Open-label, phase II study protocol	14	Cutaneous T-cell lymphoma

Discussion

Chronic pruritus is a complex skin condition that affects approximately 8% of the adult population [2], with a significant impact on the quality of life of patients. Unfortunately, there is no gold standard treatment for chronic pruritus; however, the mainstay therapy of chronic pruritus includes topical ointments such as corticosteroids, capsaicin, local anesthetics, antihistamines, immunomodulators, cannabinoids, and salicylic acid [8]. There are many different causes of pruritus, and each unique subtype of pruritus may benefit from specialized treatments. Healthcare providers should focus on the underlying cause of the pruritus and perform an extensive diagnostic workup.

Many psychiatric medications, including SSRIs, SNRIs, atypical antidepressants, and TCAs, are effective in treating chronic pruritus. Paroxetine and fluvoxamine may be beneficial in pruritus associated with atopic dermatitis, systemic lymphoma, or solid carcinoma [12]. Sertraline is particularly effective in patients with pruritus secondary to chronic liver disease [1]. Mirtazapine is an effective treatment option for nocturnal pruritus [10]. Mirtazapine can also be used to treat chronic pruritus associated with atopic dermatitis, lichen simplex chronicus, adenocarcinoma, Hodgkin’s lymphoma, large B-cell lymphoma, and advanced renal cell carcinoma that are refractory to first-line treatment [11]. Additionally, TCAs are effective for chronic pruritus. Many TCAs, including doxepin, have potent antihistamine properties. According to a systematic review of 19 clinical trials, topical low-dose doxepin was effective in treating chronic pruritus [3,14]. SNRIs may also be effective for chronic pruritus as duloxetine successfully managed multiple cases of treatment-resistant prurigo [20], and the analgesic effect of duloxetine may dull central sensitization to itch [20]; however, the scientific literature is not nearly as strong as the other classes of psychiatric medications.

Furthermore, antipsychotics may also be effective in treating specific subtypes of chronic pruritus, especially DOP. Pimozide is the treatment of choice for DOP [17]; however, studies have shown that aripiprazole and olanzapine may also be effective treatments [18].

Naltrexone, an opioid, has shown to be effective in chronic pruritus patients with cholestasis, atopic dermatitis, burns, and end-stage renal disease [27-30]. It may also be effective for nocturnal pruritus [27]. Nalmefene may be effective for patients with chronic pruritus secondary to cholestasis [31]. Intranasal butorphanol may be beneficial in patients with intractable pruritus due to dermatologic and systemic diseases [32]. It may also be beneficial for general chronic pruritus patients [33]. Because opioids have significant abuse and addiction potential, they should be used with caution in patients with pruritus.

Anticonvulsants may also be beneficial for chronic pruritus. Gabapentin is an effective therapy for chronic pruritus secondary to chronic renal failure [34]. Gabapentin and pregabalin may also be effective for chronic pruritus of unknown or unspecified origin [35,36]. Combination treatment of hydralazine and valproate may be effective in reducing pruritus in cutaneous T-cell lymphoma patients [37].

More studies on “Metaesthatic sensation” are needed for a better understanding of the spectrum of sensation ranging from light touch to severe pain. It is believed that chronic pruritus lies on this spectrum, which may explain why medications with analgesic effects can successfully treat pruritus. This concept is important as a better understanding of the itch and other sensory pathways may help develop better treatments. Additionally, many patients with pruritus may suffer from chronic pain and vice versa [5,6].

## Conclusions

With an estimated 8% of the adult population suffering from chronic pruritus, it is a common complaint. Chronic pruritus is complex and a thorough history, physical, and diagnostic workup should be done in these patients to look for the possible causes of pruritus. Chronic pruritus is caused by stimuli that are processed via somatosensory pathways that are highly individualized, creating another variable to consider when managing patient therapy. There are many different causes of pruritus. and each unique subtype of pruritus may benefit from specialized treatments. This review sheds light on the role of psychiatric, analgesic, and antiepileptic medications in chronic pruritus. In the realm of psychiatric medications, SSRIs such as paroxetine, fluvoxamine, and sertraline have been shown to be effective for multiple causes of chronic pruritus. Duloxetine, an SNRI, was found to be effective for treatment-resistant prurigo, and atypical antidepressant mirtazapine was also effective for various subtypes of chronic pruritus. Analgesic medications such as naltrexone, butorphanol, and nalfurafine have been shown to be successful in the treatment of chronic pruritus due to various causes. Anticonvulsant medications, such as gabapentin, pregabalin, and valproate, have been proven to be effective in treating certain subtypes of chronic pruritus.

However, the largest of these studies conducted for the medications mentioned in this review included 337 patients, leaving more to be desired in terms of reproducibility and reliability. Because the litany of experiments conducted is mainly small-scale, more extensive and large-scale studies are needed to determine the true effectiveness of these medications in the different subtypes of chronic pruritus. When analyzed with cautious optimism, there seems to be potential to more effectively treat this very common, yet debilitating symptom.

## References

[1] Patel T, Yosipovitch G (2010). Therapy of pruritus. Expert Opin Pharmacother.

[2] Mazeh D, Melamed Y, Cholostoy A, Aharonovitzch V, Weizman A, Yosipovitch G (2008). Itching in the psychiatric ward. Acta Derm Venereol.

[3] Steinhoff M, Cevikbas F, Ikoma A, Berger TG (2011). Pruritus: management algorithms and experimental therapies. Semin Cutan Med Surg.

[4] Stante M, Hanna D, Lotti T (2005). Itch, pain, and metaesthetic sensation. Dermatol Ther.

[5] Verhoeven EW, de Klerk S, Kraaimaat FW, van de Kerkhof PC, de Jong EM, Evers AW (2008). Biopsychosocial mechanisms of chronic itch in patients with skin diseases: a review. Acta Derm Venereol.

[6] Verhoeven EW, Kraaimaat FW, van de Kerkhof PC (2007). Prevalence of physical symptoms of itch, pain and fatigue in patients with skin diseases in general practice. Br J Dermatol.

[7] Oaklander AL (2011). Neuropathic itch. Semin Cutan Med Surg.

[8] Wang XD, Yang G, Bai Y, Feng YP, Li H (2018). The behavioral study on the interactive aggravation between pruritus and depression. Brain Behav.

[9] Buteau A, Reichenberg J (2018). Psychogenic pruritus and its management. Dermatol Clin.

[10] Hundley JL, Yosipovitch G (2004). Mirtazapine for reducing nocturnal itch in patients with chronic pruritus: a pilot study. J Am Acad Dermatol.

[11] Khanna R, Boozalis E, Belzberg M, Zampella JG, Kwatra SG (2019). Mirtazapine for the treatment of chronic pruritus. Medicines (Basel).

[12] Ständer S, Böckenholt B, Schürmeyer-Horst F, Weishaupt C, Heuft G, Luger TA, Schneider G (2009). Treatment of chronic pruritus with the selective serotonin re-uptake inhibitors paroxetine and fluvoxamine: results of an open-labelled, two-arm proof-of-concept study. Acta Derm Venereol.

[13] Mayo MJ, Handem I, Saldana S, Jacobe H, Getachew Y, Rush AJ (2007). Sertraline as a first-line treatment for cholestatic pruritus. Hepatology.

[14] Eschler DC, Klein PA (2010). An evidence-based review of the efficacy of topical antihistamines in the relief of pruritus. J Drugs Dermatol.

[15] Magazin M, Daze RP, Okeson N (2019). Treatment refractory brachioradial pruritus treated with topical amitriptyline and ketamine. Cureus.

[16] Ladizinski B, Busse KL, Bhutani T, Koo JY (2010). Aripiprazole as a viable alternative for treating delusions of parasitosis. J Drugs Dermatol.

[17] Lorenzo CR, Koo J (2004). Pimozide in dermatologic practice: a comprehensive review. Am J Clin Dermatol.

[18] Meehan WJ, Badreshia S, Mackley CL (2006). Successful treatment of delusions of parasitosis with olanzapine. Arch Dermatol.

[19] Boozalis E, Khanna R, Kwatra SG (2018). Selective serotonin reuptake inhibitors for the treatment of chronic pruritus. J Dermatolog Treat.

[20] Hashimoto T, Satoh T, Yokozeki H (2019). Prurigo successfully treated with duloxetine hydrochloride. Australas J Dermatol.

[21] Lunn MP, Hughes RA, Wiffen PJ (2014). Duloxetine for treating painful neuropathy, chronic pain or fibromyalgia. Cochrane Database Syst Rev.

[22] Sawynok J, Esser MJ, Reid AR (2001). Antidepressants as analgesics: an overview of central and peripheral mechanisms of action. J Psychiatry Neurosci.

[23] Kouwenhoven TA, van de Kerkhof PC, Kamsteeg M (2017). Use of oral antidepressants in patients with chronic pruritus: a systematic review. J Am Acad Dermatol.

[24] (2021). Pimozide | C28H29F2N3O - PubChem. https://pubchem.ncbi.nlm.nih.gov/compound/Pimozide.

[25] Rx Only ABILIFY (Aripiprazole) Tablets ABILIFY (Aripiprazole) Oral Solution CLINICAL PHARMACOLOGY Pharmacodynamics Aripiprazole Exhibits High Affinity for Dopamine D 2 and D 3, Serotonin 5-HT 1A and 5-HT 2A Receptors (K i Values of 0.34, 0.8 0.8, 1.7 1.7, and 3.4 NM, Respectively) Respectively), Moderate Affinity N H O CH 2 CH 2 CH 2 CH 2 O N N Cl Cl (2021). ABILIFY (aripiprazole) tablets. ABILIFY (aripiprazole) oral solution. https://www.accessdata.fda.gov/drugsatfda_docs/label/2005/021713s004,021436s007lbl.pdf.

[26] Umeuchi H, Togashi Y, Honda T, Nakao K, Okano K, Tanaka T, Nagase H (2003). Involvement of central mu-opioid system in the scratching behavior in mice, and the suppression of it by the activation of kappa-opioid system. Eur J Pharmacol.

[27] Mansour-Ghanaei F, Taheri A, Froutan H (2006). Effect of oral naltrexone on pruritus in cholestatic patients. World J Gastroenterol.

[28] Malekzad F, Arbabi M, Mohtasham N (2009). Efficacy of oral naltrexone on pruritus in atopic eczema: a double-blind, placebo-controlled study. J Eur Acad Dermatol Venereol.

[29] Jung SI, Seo CH, Jang K, Ham BJ, Choi IG, Kim JH, Lee BC (2009). Efficacy of naltrexone in the treatment of chronic refractory itching in burn patients: preliminary report of an open trial. J Burn Care Res.

[30] Peer G, Kivity S, Agami O, Fireman E, Silverberg D, Blum M, laina A (1996). Randomised crossover trial of naltrexone in uraemic pruritus. Lancet.

[31] Bergasa NV, Alling DW, Talbot TL, Wells MC, Jones EA (1999). Oral nalmefene therapy reduces scratching activity due to the pruritus of cholestasis: a controlled study. J Am Acad Dermatol.

[32] Dawn AG, Yosipovitch G (2006). Butorphanol for treatment of intractable pruritus. J Am Acad Dermatol.

[33] Kumagai H, Ebata T, Takamori K, Muramatsu T, Nakamoto H, Suzuki H (2010). Effect of a novel kappa-receptor agonist, nalfurafine hydrochloride, on severe itch in 337 haemodialysis patients: a Phase III, randomized, double-blind, placebo-controlled study. Nephrol Dial Transplant.

[34] Gunal AI, Ozalp G, Yoldas TK, Gunal SY, Kirciman E, Celiker H (2004). Gabapentin therapy for pruritus in haemodialysis patients: a randomized, placebo-controlled, double-blind trial. Nephrol Dial Transplant.

[35] Yesudian PD, Wilson NJ (2005). Efficacy of gabapentin in the management of pruritus of unknown origin. Arch Dermatol.

[36] Ehrchen J, Ständer S (2008). Pregabalin in the treatment of chronic pruritus. J Am Acad Dermatol.

[37] Espinoza-Zamora JR, Labardini-Méndez J, Sosa-Espinoza A (2017). Efficacy of hydralazine and valproate in cutaneous T-cell lymphoma, a phase II study. Expert Opin Investig Drugs.

